# Underestimated Fatalities of a Cryptic Avian Species of Conservation Concern at Wind Energy Facilities in California, USA

**DOI:** 10.1002/ece3.72855

**Published:** 2026-01-26

**Authors:** Todd E. Katzner, Ashley M. Spicer, Patricia A. Ortiz, Tara J. Conkling

**Affiliations:** ^1^ U.S. Geological Survey, Forest and Rangeland Ecosystem Science Center Boise Idaho USA; ^2^ School of Natural Resources and the Environment West Virginia University Morgantown West Virginia USA; ^3^ California Department of Fish and Wildlife Law Enforcement Division Sacramento California USA; ^4^ U.S. Fish and Wildlife Service, Migratory Birds and Habitat Program Portland Oregon USA

**Keywords:** *Agelaius tricolor*, cryptic species, forensic genetics, Icteridae, misidentification, tricolored blackbird, wind turbines

## Abstract

Accurate information underpins successful ecological science and management. Cryptic species, those that are difficult to differentiate, pose challenges to reliable collection of taxon‐specific information. Blackbirds, including tricolored blackbirds ( *Agelaius tricolor* ), a cryptic species of high conservation concern, and red‐winged blackbirds ( *Agelaius phoeniceus* ), an abundant congener, are sometimes killed by wind turbines. We used publicly available survey records to evaluate rates at which blackbirds were reported dead at wind energy facilities in California, USA. We then used genetic species identification of carcasses found to estimate true rates of discovery and of misidentification. Of 329 blackbird fatalities in survey records, most were identified as red‐winged (*n* = 149), “unidentified” (*n* = 90), or Brewer's ( *Euphagus cyanocephalus* ; *n* = 70); only 13 were identified as tricolored. We also genetically analyzed samples from 40 blackbirds. Of 14 carcasses identified in the field to species, two, including one tricolored, were incorrectly called Brewer's blackbirds (14% misidentification rate). Of the 26 birds called “unidentified blackbird” in the field, 17 (65%) were tricolored, leading to a 19× underestimation of true fatality rate. The state‐wide population of tricolored blackbirds is < 1% the size of that of red‐winged blackbirds. A large proportion of blackbirds found dead were actually tricoloreds, indicating that fatality rates of this state threatened species may be substantially underestimated. The potential for misidentification or nonidentification may create perverse incentives that undermine conservation and have consequences for on‐the‐ground management, mitigation, and operations of high‐priority infrastructure.

## Graphical abstract

Blackbirds killed at wind turbines in California, USA, are typically assumed to be common red‐winged blackbirds. In reality, a surprising number of them are endangered tri‐colored blackbirds. This finding has important implications for the way fatalities are managed at these wind facilities.

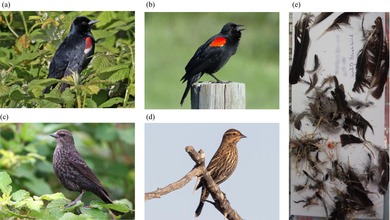

## 1 Introduction

Conservation depends on reliable identification and counts of flora and fauna (Bickford et al. 2007). Typically, expert opinion, field guides, and specialized tools help to achieve this goal. However, cryptic species, those with phenotypic characteristics so similar they are difficult to differentiate, pose challenges to science and management globally (Hebert et al. 2004; Cook et al. 2008; Trontelj and Fišer 2009; Delić et al. 2017; Struck et al. 2018; Yan et al. 2018). In fact, misidentification of, or even failure to identify, cryptic species can have dramatic impacts in conservation settings, affecting distributional models (Aubry et al. 2017), disease transmission (Zhong et al. 2020), and the design of potential mitigation actions (Gruppi et al. 2023). As such, documenting misidentification or nonidentification of cryptic species is essential because recognition of the problem can alter ongoing management.

Resource management has emerged as a top priority at renewable energy facilities. This has occurred because renewable energy is expanding at a rapid pace globally, often as one component of a strategy to reduce carbon emissions (IPCC 2014; Pörtner et al. 2021). Nevertheless, there are environmental problems that may stem from renewable energy (Allison et al. 2019). Renewable energy development can alter landscapes or weather and kill or injure wildlife (Katzner et al. 2025; Kosciuch et al. 2020; Walston Jr. et al. 2016). In the case of wildlife, the most visible impacts are in the form of fatalities, typically from collision with energy‐generating machinery or associated infrastructure. Surveys regularly are implemented to count killed or injured individual animals at renewable energy facilities (Conkling et al. 2020), and these data are sometimes used to assess demographic impacts (Conkling et al. 2022). However, these assessments rely on the assumption of accurate species identification. Misidentification of cryptic species would undermine that assumption and thus lessen the value and effectiveness of those surveys for understanding impacts of renewables on wildlife and for guiding mitigation.

**FIGURE 1:**
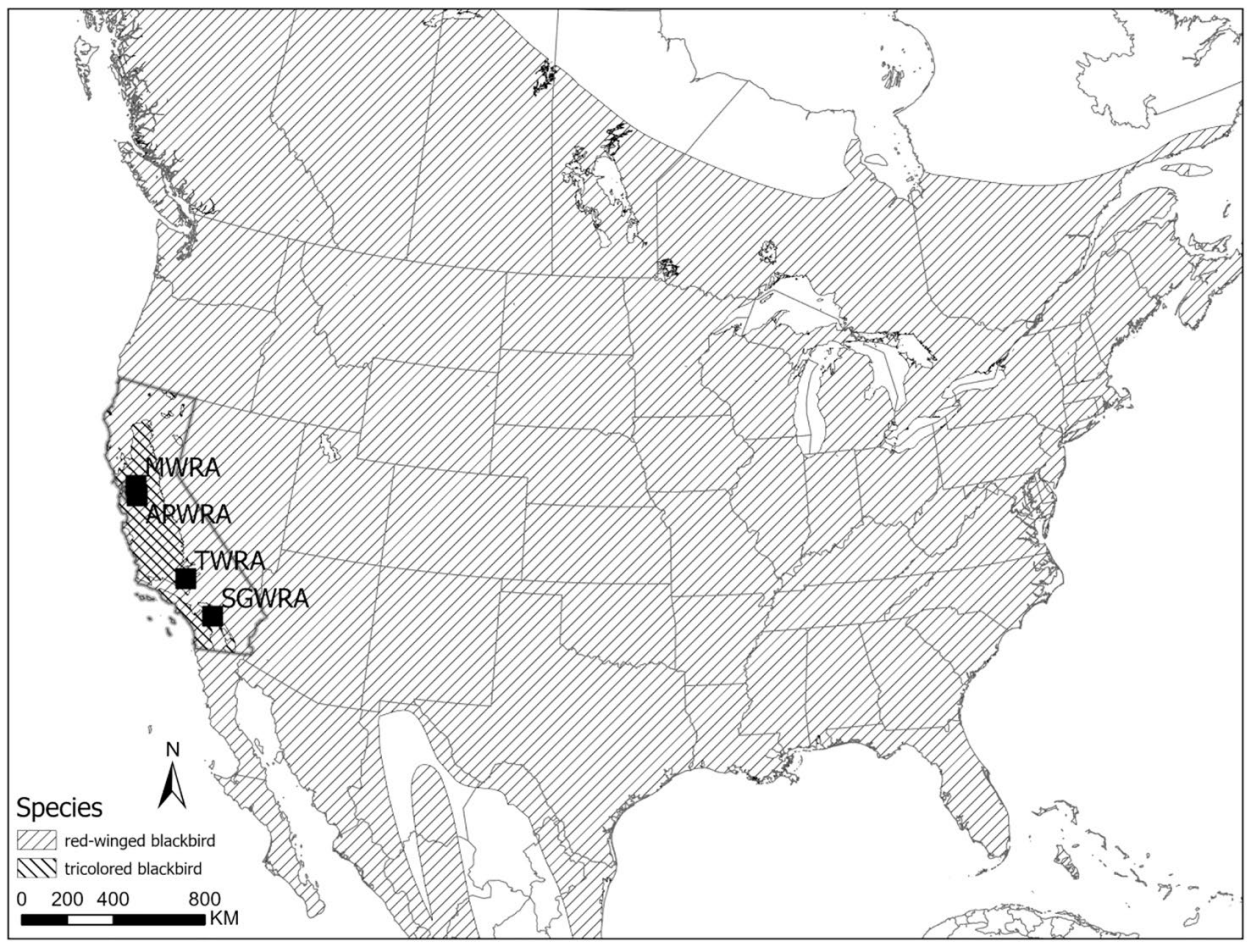
Map showing the continent‐wide distribution of red‐winged blackbird and the range‐restricted distribution of tricolored blackbird. Also shown are the locations of the four wind resource areas considered in this study. APWRA, Altamont Pass Wind Resource Area; MWRA, Montezuma Hills Wind Resource Area; SGWRA, San Gorgonio Wind Resource Area; TWRA, Tehachapi Wind Resource Area (includes one adjacent facility). Map data are from Natural Earth (www.naturalearthdata.com). Range map for red‐winged blackbird is from Birdlife International and Handbook of the Birds of the World (2024) and for tricolored blackbird from California Department of Fish and Wildlife California Interagency Wildlife Task Group (CDFW‐IWTG) (2016).

**FIGURE 2:**
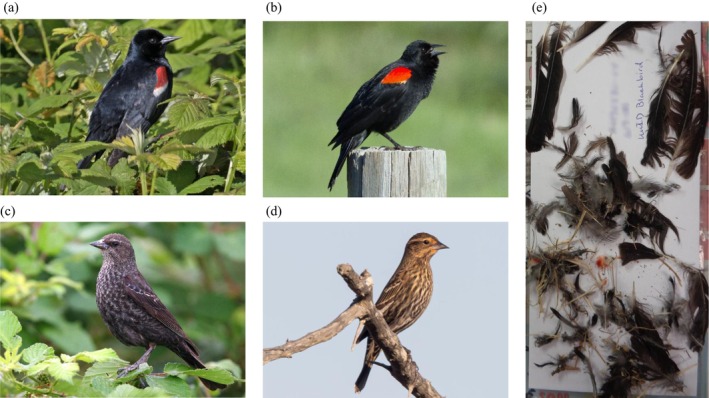
Cryptic blackbirds, including a (a) male tricolored blackbird; (b) male red‐winged blackbird, (c) female tricolored blackbird, and (d) female red‐winged blackbird, (e) remains of a tricolored blackbird described in the field as an “unidentified blackbird” when found dead at the Altamont Pass Wind Resource Area (APWRA). Photos by T. Beedy (a–c), T. Katzner (d), US Geological Survey (e).

In the state of California, there are two closely related, co‐occurring, and cryptic species of blackbirds (Family Icteridae), the abundant red‐winged blackbird ( *Agelaius phoeniceus* ; North American population size = ~180 million; Will et al. 2020; Yasukawa and Searcy 2020) and the at risk and rapidly declining tricolored blackbird ( *Agelaius tricolor* ; global population size = ~180,000; Beedy et al. 2023; BirdLife International 2020; Airola et al. 2024; California Natural Diversity Database (CNDDB) 2024). Red‐winged blackbirds are distributed across North America and into central America, while tricoloreds have a distribution limited primarily to central and southern California, with a few breeding populations in Oregon and Washington (Figure 1). Males of the two species are similar when in breeding plumage, but differentiating them is straightforward (Figure 2a,b). However, females, juveniles, and nonbreeders can be extremely difficult to differentiate (Figure 2c,d; Beedy et al. 2023; Yasukawa and Searcy 2020). Differentiating the two species can be especially difficult when degraded carcasses or feather spots (Figure 2e) are found in the field. In these situations, specimens of both species also can be confused with similarly sized, widespread, and abundant Brewer's blackbird ( *Euphagus cyanocephalus* ; North American population size = ~23 million; Martin 2020; Will et al. 2020) or brown‐headed cowbirds ( *Molothrus ater* ; North American population size = ~130 million; Lowther 2020; Will et al. 2020).

Blackbird carcasses, often in poor condition, have been recovered at wind turbines in California, USA. Because one of these species is protected by the California Endangered Species Act (CESA; Cal. Fish & G. Code § 2050), misinformation in survey reports can have substantial consequences for conservation and management. For example, CESA prohibits “take” of protected species without authorization but also authorizes the California Department of Fish and Wildlife (CDFW) to permit incidental take, typically when accompanied by minimization or mitigation actions (CDFW 2025). Penalties for noncompliance can be substantial, and thus both regulators and potential permittees have a strong interest in accurate identification of potential take.

We were interested to understand if and how fatalities of these cryptic species may be conflated at wind facilities in the California part of their range where the distribution of the two species overlaps. To do this, we used (1) literature review of field‐based surveys to understand reported rates of discovery of dead blackbirds of each species at wind energy facilities; and (2) genetic analyses of carcasses found at wind facilities to estimate rates of misidentification and true rates of discovery. Taking these two steps allows us to understand the rate at which misidentification may occur relative to the total scale of blackbird fatalities at wind facilities. Finally, we discuss our results in the context of misinformation from surveys at wind facilities and management of cryptic species in general.

## 2 Methods

### 2.1 Literature Review: Rates of Discovery of Dead Blackbirds

We searched an existing database of environmental reports describing field surveys at renewable energy facilities (Conkling et al. 2020). This database was previously compiled from California‐specific federal, state, and county‐level agencies, and other publicly available document collections. Details on construction of the original database, how data are summarized, and how reports may be accessed are described in Conkling et al. (2020). For the present analysis, we reviewed the subset of postconstruction reports, field datasets, and peer‐reviewed literature produced through March 2024 from surveys at wind facilities at four wind resource areas within the range of tricolored blackbirds (as delineated by California Department of Fish and Wildlife California Interagency Wildlife Task Group (CDFW‐IWTG) 2016; Figure 1). We removed from consideration those reports that duplicated other reports in our collection (e.g., we removed monthly reports if there was also an annual report for the same facility in the same year).

We used these reports to summarize, by species, the frequency with which fatalities (both from surveys and incidental finds) were reported for brown‐headed cowbird, red‐winged blackbird, Brewer's blackbird, tricolored blackbirds, yellow‐headed blackbird ( *Xanthocephalus xanthocephalus* ), and “unidentified” blackbirds (those carcasses not identified to species, but broadly classified as blackbirds). Cowbirds are in the Family Icteridae and can be confused with a blackbird; thus, for the purposes of this study, we consider them here as “blackbirds.” For the subset of reports that assigned individual observations to specific dates, we summarized fatalities by year and month.

We used a two‐sided Fisher's exact test and pairwise comparisons with adjusted *p* values (*α* = 0.05) in R (fisher.test, fisher.multcomp; R Core Team 2022, Hervé 2023) to test for monthly differences in counts by species and annual differences in the number of identified versus unidentified blackbirds reported found. All reports we evaluated provided counts of birds found, but only some adjusted those totals by searcher efficiency or carcass persistence. Consequently, here we report and analyze only raw totals (i.e., we ignore corrections for detection rates), and we draw inference only to those unadjusted data.

### 2.2 Field Data: Rates of Misidentification and True Rates of Discovery

#### 2.2.1 Field Sample Collection

We obtained feather samples from fatalities of blackbirds collected from 2011 to 2019 at multiple wind facilities in the Altamont Pass Wind Resource Area (APWRA), located in Alameda and Contra Costa Counties, California. Fatalities considered in this study were found incidentally or during systematic surveys and consisted of whole or partial carcasses whose condition ranged from fresh to desiccated, decomposed, or feather spots (Figure 2e). Almost all fatalities from which we collected samples were initially documented in the reports noted above, although a few of the carcasses we sampled were not in reports available to us. Likewise, because this study was designed long after most of the field surveys were conducted, only a subset of the carcasses found were retained and thus available for sampling and genetic analyses. After collection, field crews identified each carcass to species as accurately as possible in the field, and then archived the remains in designated on‐site freezers. Subsequently, carcasses or feather spots were transported to the lab, photographs were taken, and representative tissue and feather samples were collected and then stored frozen or dried.

#### 2.2.2 Laboratory Genetics

We used forensic genetic techniques to confirm the species identification for all archived carcasses or feather spots that had been identified in the field as blackbirds. Our analysis approach involved amplification and sequencing of the mitochondrial NADH dehydrogenase subunit 2 (ND2) gene region (449–550 base pairs; bp). For degraded samples, a shorter ND2 amplicon (201 bp) was sequenced to confirm the correct species (for additional details, see Supporting Information: Lab Methodology: Primer design).

We extracted DNA from feathers and, in one case, bone (for additional details, see Supporting Information: Lab Methodology: DNA extraction). We amplified the resulting genetic material using a combination of published primer sequences and the designed primer (Table S1). Sequences were evaluated for quality, edited, aligned, and compared to reference sequences for species identification (Supporting Information: Lab Methodology: Sanger sequencing and analysis).

## 3 Results

### 3.1 Literature Review: Rates of Discovery of Dead Blackbirds

Our literature database contained 56 postconstruction reports and datasets covering avian fatalities from 1997 to 2023 at wind facilities within the range of tricolored blackbirds. After removing reports that contained similar information, we ultimately considered data from 22 reports (Table 1). These were either from stand‐alone facilities or from multiple facilities at one of four wind resource areas within California (Figure 1).

**TABLE 1:** Uncorrected (raw) fatality aggregate totals gathered from 22 publicly available survey reports and datasets of blackbirds found dead at a subset of California, USA, wind facilities or wind resource areas from 1998 to 2023 (a 23rd report referenced in the main text was used to provide temporal information for Figure S1, but the information it contained is covered in other reports on this list). Details on accessing these reports are provided in the main text and in Conkling et al. (2020). Because of the broad time range, wind facilities include turbines of many sizes and generations. APWRA, Altamont Pass Wind Resource Area; MWRA, Montezuma Hills Wind Resource Area; SGWRA, San Gorgonio Wind Resource Area; TWRA, Tehachapi Wind Resource Area (includes one adjacent facility). Data here are reports on 331 dead blackbirds (the first six columns of the table) and also include the 287 dead birds for which date information were sufficient for the monthly and annual analyses shown in Figure 3 and Figure S1. Numbers of samples used in genetic analyses from each report or time period are indicated in parentheses. Two red‐winged blackbird carcasses we genetically analyzed were found outside of the time periods covered by the reports in this table and so only 38 genetically analyzed individuals are noted here.

Wind resource area	Time period	Brown‐headed cowbird	Brewer's blackbird	Red‐winged blackbird	Tricolored blackbird	Yellow‐headed blackbird	Unidentified blackbird	Unidentified small bird
APWRA	1998–2003	2	13	12	1	—	1	42
	2005–2013	1	18	28 (2)	3	—	44	233
	2008–2011	—	3	—	—	—	—	—
	2012–2015	—	1	3	1	—	1	8
	2016–2019^a^	1 (1)	4 (4)	1 (1)	4 (1)	—	22 (21)	52
	2019–2022	—	1 (1)	2	—	—	—	12
	2023^b^	—	—	—	1	—	1	7
	2018‐2021^a^	—	1 (1)	1 (1)	1	—	5 (5)	57
	2023^b^	—	—	—	—	—	1	10
	2021–2022	—	1	—	—	—	—	8
	2022–2023	—	—	1	—	—	—	9
MWRA	2003–2005	—	2	14	—	—	1	1^c^
	2011–2012	—	1	16	—	—	7	18
	2012–2013	—	—	1	—	—	2	—^d^
	2006–2009	—	15	47	2	—	3	1^c^
	2009–2010	—	—	5	—	—	1	5^c^
	2009–2012	—	4	15	—	—	1	—^d^
	2012–2013	—	4	4	—	—	—	—^d^
	2012–2013	1	1	1	—	—	—	1
SGWRA	1997–2000	—	1	—	—	—	—	18^c^
TWRA	2017–2018	—	—	—	—	1	—	—
	2015–2016	—	—	—	—	1	—	7
Totals	1997–2021	5	70	151	13	2	90	489

^a^ Annual summary totals reported in the main text of this report differed from individual records in the appendices, so we report the largest fatality total provided.

^b^ No avian fatality monitoring. Birds were found dead during surveys for bat carcasses.

^c^ Value is only for “Unidentified bird” since monitoring efforts in this report did not distinguish unidentified fatalities based on size class.

^d^ Report included no “unidentified birds” (all carcasses classified to species or taxonomic group).

These reports contained data on 331 fatalities of blackbirds (Table 1). Of these, the most common were red‐winged (*n* = 151), unidentified (*n* = 90), and Brewer's (*n* = 70) blackbirds. Tricolored blackbirds were only rarely reported (*n* = 13). The majority of reports (15 of 22) made no mention of observations of tricolored blackbirds, alive or dead.

**FIGURE 3:**
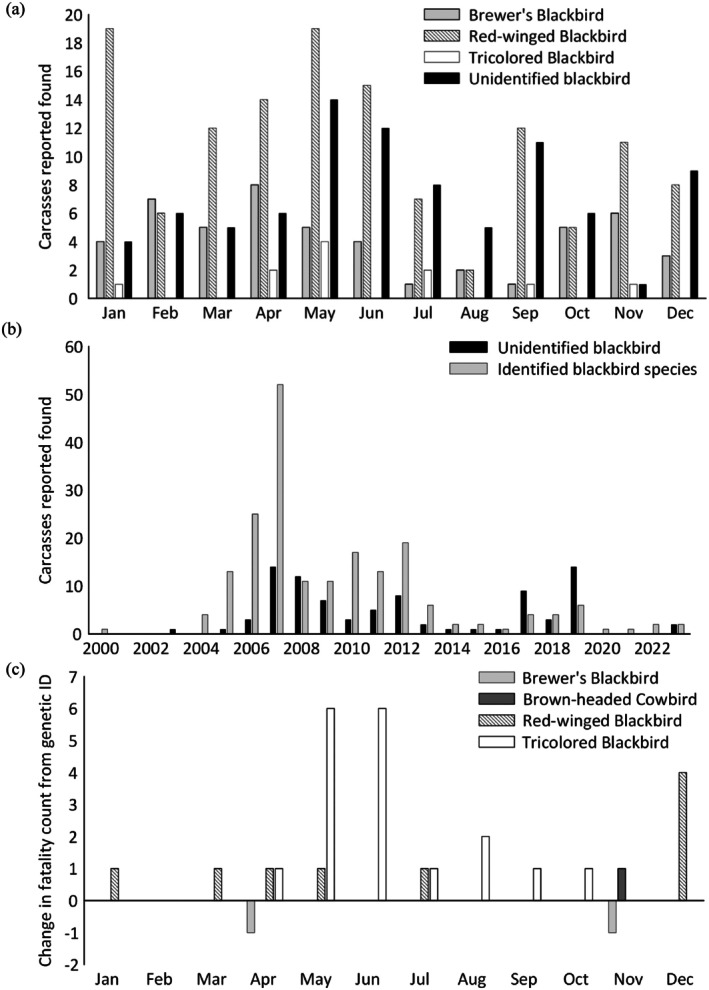
Numbers of carcasses, including feather spots, of blackbirds and cowbirds reported from wind energy facilities in California, USA during the period 1998–2023. Plots show (a) carcasses reported found per month for the three most common species at four wind resource areas; (b) the numbers of blackbirds and cowbirds identified to species and those identified as “unidentified blackbird,” by year at four wind resource areas (*x*‐axis excludes 1998–1999, during which no blackbird carcasses were found); and (c) change in numbers of fatalities as a consequence of genetic identification (*n* = 40 carcasses from a single wind resource area), organized by month and by species. Not shown on plots (a) and (b) are rarely found species, specifically, two brown headed cowbirds (one each found in May and in September) and two yellow‐headed blackbirds (April and May).

Records for 287 fatalities from 21 of the reports contained date information that could be used analysis of monthly and annual patterns (Figure S1). The species composition of blackbird carcasses reported varied by month (Figure 3a; *p* = 0.029). Although we detected no pairwise differences (*p* > 0.05), the greatest absolute counts of unidentified blackbird carcasses were reported in May, June, September, and December. The number of unidentified blackbirds reported also varied with year (Figure 3b; *p* < 0.001). This difference was driven primarily by fatality monitoring in 2006 and 2007 at both Montezuma Hills Wind Resource Area (MHWRA) and APWRA that classified most blackbird fatalities as red‐winged or Brewer's (*p*_2019:2006_ = 0.008, *p*_2019:2007_ = 0.023; Table 1, Figure 3), a pattern that did not continue throughout all later years. Rates of identification also appeared to vary by location, with unidentified classifications making up < 10% of blackbirds (*n* = 13) found at MHWRA, but nearly half (49%, *n* = 74) at APWRA.

### 3.2 Field Data: Rates of Misidentification and True Rates of Discovery

Our working collection contained samples from remains of 40 blackbirds, of which 20 were described as feather spots, originating from APWRA (Table 1). These had been identified in the field as brown‐headed cowbird (*n* = 1; 3%), and Brewer's (6; 15%), red‐winged (6; 15%), tricolored (1; 3%), and “unidentified” (26; 65%; 19 feather spots) blackbirds (Table 2).

**TABLE 2:** Field versus genetic identification of 40 blackbirds and cowbirds found dead at the Altamont Pass Wind Resource Area (APWRA), California, USA. Also shown are proportions and numbers of each species correctly identified in the field and the correction factor implied by the genetic analysis of identified + unidentified individuals.

Species	Field ID	Genetic ID	Correct field ID^a^	Correction factor^b^
Brewer's blackbird	6	4	67% (1)	0.67
Brown‐headed cowbird	1	2	100% (1)	2.00
Red‐winged blackbird	6	15	100% (6)	2.50
Tricolored blackbird	1	19	100% (1)	19.00
Unidentified blackbird	26	—	0% (26)	—
Yellow‐headed blackbird^c^	—	—	—	

^a^ “Correct field ID” was calculated for each species as (# correctly identified/[# correctly identified + # incorrectly identified]) and *does not* include birds identified as “unidentified.”

^b^ Correction factor was calculated for each species as (# identified genetically/# identified in the field) and *does* include birds identified as “unidentified.”

^c^ Although none were identified genetically, yellow‐headed blackbirds are included in the table because they were reported in our review of field survey data, and we genetically evaluated their potential presence (Table S1).

Genetic analyses of identified and unidentified carcasses suggested that counts of all species were incorrect. True numbers of Brewer's blackbird were 33% lower than indicated in field tallies. Numbers of all other species increased over field tallies by factors of 2–19, and the carcasses we considered were composed predominantly of at risk tricolored blackbirds. Of the 26 blackbirds called “unidentified” in the field, the vast majority were actually tricolored (*n* = 17; 65%). The remainder were red‐winged (9; 35%). Correct counts of all species included 19 tricoloreds (48%), 15 red‐winged (38%), four Brewer's (10%), and two cowbirds (5%).

Species determinations were correct for 12 of 14 individuals identified in the field (86%). These included four of six Brewer's blackbirds (67%; one was in fact a cowbird, the other a tricolored blackbird), all six red‐winged blackbirds, the tricolored blackbird, and the brown‐headed cowbird.

Use of genetic identification tools changed counts of fatalities in 11 of the 12 months of the year (Figure 3c). Unidentified blackbirds that were later genetically identified as tricolored were found only from April to October. Unidentified blackbirds that were later genetically identified as red‐winged were found from December to July. The two birds misidentified as Brewer's blackbirds were found in April (a tricolored blackbird) and November (a cowbird).

## 4 Discussion

Surveys for wildlife affected by development are a critical component of environmental and regulatory management. Our work shows that despite expert effort, these surveys sometimes produce misinformation that can have large‐scale consequences. In this case reported rates of discovery of a cryptic species of high conservation concern were off by 19×, an error rate that has substantial consequences given the large number of blackbird fatalities reported over time. Estimated mortality rates appear to have been conflated primarily by nonidentification and, to a lesser degree, by misidentification. Although our identification data and misclassification rate are from one wind resource area, this work has implications not only for species‐based conservation but also for mitigation and management of a state‐listed threatened species.

### 4.1 Conflation of Cryptic Species and Conservation of Tricolored Blackbirds

Because the size of the tricolored blackbird population in California is ~1% of the size of the red‐winged population in the state (Will et al. 2020), it is not surprising that when blackbird carcasses are found, they are most often identified as red‐winged. It was therefore unexpected that (a) the majority of blackbirds we genetically identified were in fact tricoloreds, (b) the greatest discrepancy between reported and true rates of discovery was for tricoloreds, and (c) 65% of unidentified blackbirds were in fact tricolored.

If the patterns we identified hold for all unidentified blackbirds found dead at APWRA, or at other wind energy facilities, then an unexpectedly large number of individuals of this species are killed each year by wind turbines. In one scenario, estimates of tricolored fatalities could be off by a factor of 19. Alternatively, if 65% of the 90 unidentified blackbirds described in field reports were actually tricolored, it would represent 4.5× more tricoloreds than documented in those reports. Even the smaller of these two estimates would have implications for conservation management.

It is notable that there are regional differences in frequency of species identification and that unidentified birds are more commonly reported now than during earlier years. Given these increasing numbers of reported unidentified birds, it seems possible that, in earlier surveys, a number of the blackbirds identified as red‐winged were tricolored. It is plausible also that the 2019 change in conservation status of the tricolored blackbird (to “threatened”) may have encouraged field surveyors to be more cautious in species identification (CNDDB 2024).

Tricolored blackbirds are experiencing substantial population declines, and thus are of high conservation concern (BirdLife International 2020; CNDDB 2024). Although habitat loss and pesticide use, both leading to breeding failure, are thought to be primary causes of decline, collision through fatality is considered relevant (Beedy et al. 2023). Without the genetic analyses we performed, it would have been reasonable to infer that the 13 individuals noted in reports were an accurate approximation of the numbers actually killed. However, such inference would have been incorrect, a finding particularly relevant to any mitigation strategies developed in response to these fatalities.

### 4.2 Cryptic Species and Conservation

Cryptic species present substantial difficulties for monitoring, whether for basic or applied ecological studies. In the case of renewable energy facilities such as those we studied, surveyors sometimes encounter species that can be difficult to differentiate without modern technological tools. Consequently, when there are questions about identification, field surveyors may often call fatalities “unidentified.” In our work, two factors likely further confused inference. First, different corporations, presumably with different experts and different perspectives on uncertainty, performed these surveys and identified the carcasses. Second, early surveys at these wind facilities were different than they are more recently. Specifically, early surveys used human searchers, they were conducted at intervals of seven to 40 days, and they targeted raptors. Some of the more recent surveys were at shorter intervals and relied on a new technique, using dog‐handler teams to search for fatalities. There is abundant evidence that dogs are far more effective than humans at finding wildlife carcasses (Domínguez del Valle et al. 2020; Grimm‐Seyfarth et al. 2021), and dogs often find small parts of birds or a small group of feathers from a single individual (a feather spot). Finding feather spots may result in more reports of unidentified species because the feather spots may have insufficient distinguishing characteristics to resolve identification, not only within a taxonomic group but sometimes even across groups. For example, in a recent study, a feather spot described in the field as being from a “small bird” was genetically identified as an eared grebe ( *Podiceps nigricollis* ), and a feather spot from an “unidentified blackbird” was genetically identified as a western kingbird ( *Tyrannus verticalis* ; Gruppi et al. 2023). We have no data on how frequently the 489 unidentified small birds in Table 1 were actually tricolored blackbirds. Thus, implementation of these new field techniques with dogs has led to increased collection of feather spots and partial carcasses, which in turn increases reliance on more robust methods (genetics or even microscopy; Dove and Koch 2011) to obtain accurate identification of cryptic species.

Misinformation can be a substantial threat to conservation, in this case potentially underestimating the degree of threat posed to a state threatened species. Furthermore, the cryptic nature of these blackbirds may create perverse incentives to perpetuate misinformation. Mitigation for anticipated take is expensive and penalties for noncompliance with CESA can be substantial. Stakeholders wishing to avoid these costs could be incentivized to report unidentified birds instead of accurately identifying carcasses. In the absence of genetic analyses such as we performed, a reasonable default for managers would have been to assume that most dead blackbirds are red‐winged. In such a scenario, all parties are identifying species to the best of their ability, yet the hidden perverse incentive perpetuates misinformation, potentially undermining conservation (Langpap and Wu 2017).

It is impossible to evaluate the relevance of conservation measures to support cryptic species such as tricolored blackbirds if the species are not correctly identified. Correct identification in the modern world relies on technological solutions such as the genetic tools we applied here and reduces scientific uncertainty and misinformation, leading to more informed policy outcomes from decision‐makers. For example, in a case such as this, correct information would allow a better understanding of the true demographic impacts of wind energy on this state‐listed species, provide accurate information to guide the design and implementation of mitigation programs, and influence the operations of high‐priority infrastructure. This work therefore illustrates the conservation value of efforts to identify cryptic species that may be impacted by anthropogenic stressors.

## Author Contributions

**Todd E. Katzner:** conceptualization (equal), data curation (equal), formal analysis (equal), funding acquisition (equal), investigation (equal), methodology (equal), project administration (equal), resources (equal), supervision (equal), validation (equal), writing – original draft (equal), writing – review and editing (equal). **Ashley M. Spicer:** conceptualization (equal), data curation (equal), formal analysis (equal), investigation (equal), writing – review and editing (equal). **Patricia A. Ortiz:** conceptualization (equal), data curation (equal), investigation (equal), project administration (equal), writing – review and editing (equal). **Tara J. Conkling:** conceptualization (equal), data curation (equal), formal analysis (equal), investigation (equal), writing – review and editing (equal).

## Funding

This work was supported by the author's institutions.

## Conflicts of Interest

The authors declare no conflicts of interest.

## Supporting information


**Data S1:** ece372855‐sup‐0001‐Supinfo.docx.

## Data Availability

All data associated with this work have been deposited in public databases (genetic sequences) or are presented in the manuscript.
