# Supplementary material for: Underestimated Fatalities of a Cryptic Avian Species of Conservation Concern at Wind Energy Facilities in California, USA
Source: Ecol Evol. 2026 Jan 26;16(1):e72855. doi: 10.1002/ece3.72855 (PMC12834644; doi:10.1002/ece3.72855)
Supplement: Supplementary file 1 — Data S1: ece372855‐sup‐0001‐Supinfo.docx. [file ECE3-16-e72855-s001.docx]

**Supplementary Materials:**

**Underestimated fatalities of a cryptic avian species of conservation concern at wind energy facilities in California, USA**

**Todd E. Katzner^aǂ^, Ashley M. Spicer^b^, Patricia A. Ortiz^a#^, Tara J. Conkling^a^**

**^a^ U.S. Geological Survey, Forest and Rangeland Ecosystem Science Center, 230 N. Collins Rd., Boise, ID, 83702, USA**

**^b^ California Department of Fish and Wildlife, Law Enforcement Division, Sacramento, CA, USA**

**^ǂ^ Correspondence:** [**tkatzner@usgs.gov**](mailto:tkatzner@usgs.gov)

**^#^ Present address: US Fish and Wildlife Service, Migratory Birds and Habitat Program**

**Lab Methodology**

*Primer design*

To identify low-content or highly degraded DNA samples, we used Primer3 version 4.1.0 and the NCBI BLAST program to design a reverse primer to produce a short ND2 amplicon (201 bp) with sufficient inter-species variability to distinguish the avian species of interest in this study. The 201 bp ND2 fragment is relative to nucleotide positions 5217-5417 of the red junglefowl, *Gallus gallus,* GenBank accession number X52392.1 (Desjardins and Morais 1990).

Tricolored blackbirds are considered sister species to the closely related red-winged blackbird and the red-shouldered blackbird (*Agelaius assimilis*). The red-shouldered blackbird was not included in the primer design as this species is endemic to Cuba and thus outside the geographic scope of consideration for this study. Phylogenetic analyses of tricolored and red-winged blackbird mtDNA have demonstrated fixed haplotype differences in the ND2 gene region between these two species (Barker et al. 2012). Because we used mitochondrial DNA, our approach assumes no hybridization between the different species we considered (none has been detected for the two *Agelaius* species; Beedy et al. 2023).

We used GenBank sequences of tricolored blackbird (GenBank accession numbers AF109949.1 and JX512634.1; Johnson and Lanyon 1999 and Barker et al. 2012, respectively) and of red-winged blackbird (JX512632.1 and NC_018801.1; Barker et al. 2012 and Powell et al. 2013, respectively). Subsequently, primer specificity was tested using in-house references (Table S2) and GenBank sequences for Brewer’s blackbird (AF109951.1; Johnson and Lanyon 1999), brown-headed cowbird (MN356438.1; Feng et al. 2020), yellow-headed blackbird (KC007584.1; Barker et al. 2013), and rusty blackbird (*Euphagus carolinus*; AF109950.1; Johnson and Lanyon 1999). Yellow-headed blackbird is uncommon at APWRA and rusty blackbird has not been reported (eBird 2021), but both were included for confirmatory purposes. Primers were manufactured by ThermoFisher Scientific, High Point, NC.

Using this distance-based approach for species identification required assessing that the intraspecific variability did not overlap with the interspecific variability for the ND2 amplicon (Meiklejohn et al. 2021; Table S3).

*DNA extraction*

We removed approximately 0.5 cm-1.0 cm of the proximal end of the calamus, or quill, of the feather with a sterile razor and placed with clean forceps into a 1.5 mL sample tube. We did not clean the sampled surface area prior to sampling, in order to preserve any dried blood or tissue that was present on the sampled region and putatively deposited at the time of death. We digested feather samples in 400 µL of a dithiothreitol (DTT)-based digestion buffer (78 mM DTT; 100 mM NaCl; 0.5 mg/mL Proteinase K; 2 g/mL SDS; 3 mM CaCl_2_; and TE Buffer, pH 8.0) and then incubated them for 24 hours at 56 °C at 1000 rpm on an Eppendorf Thermomixer® C (Eppendorf AG). After digestion, we centrifuged the samples at 15,000 rpm for 90 seconds to pellet any undissolved tissues along with pigments that may have been present in the feather samples. We removed the supernatant in 200 µL aliquots and added to two separate 2.0 mL EZ1 sample tubes. DNA was extracted using a Qiagen EZ1® Advanced XL DNA extraction robot (Qiagen, Inc.) following manufacturer’s protocol for EZ1 DNA Investigator trace samples and eluted in 50 µL of water. We combined eluates from the two DNA split samples for a total of 100 µL of DNA elution volume per sample. We performed sampling, extraction, amplification, and sequencing steps in separate rooms with designated work areas, PCR hoods, pipettes, and reagents to prevent cross-contamination.

*Sanger sequencing and analysis*

We amplified avian ND2 sequences in a 25 µL reaction volume containing 2.5 µL 10X PCR buffer (Applied Biosystems^TM^), 1.3 µL dNTP mix (2.5 mM each), 0.5 µL forward and reverse primers (10 µM each) tagged with M13F or M13R, respectively, 2.0 µL MgCl_2_ (Applied Biosystems^TM^), 0.4 µL BSA (10 mg/mL), 0.26 µL AmpliTaq Gold^TM^ (Applied Biosystems^TM^), 2.5 µL template DNA, and PCR-grade water to volume. Thermocycling parameters were as follows: 95 °C for 10 min, followed by 35 cycles of 95 °C for 60 sec, 54 °C for 60 sec, 72 °C for 60 sec, and a 5 min final extension at 72 °C. We purified products using ExoSAP-IT^TM^ PCR Product Cleanup Reagent (Applied Biosystems^TM^) Low Volume protocol in a 5 µL reaction volume containing 0.40 µL ExoSAP-IT^TM^, 4.60 µL Tris Buffer, pH 8.0 (50 mM), and 1-2 µL of PCR amplification product with thermocycling conditions of 37 °C for 30 min, followed by 85 °C for 15 min. Purified amplification products were sequenced using the BigDye^TM^ Terminator v3.1 Cycle Sequencing Kit (Applied Biosystems^TM^) with M13F and M13R primers and purified using the BigDye XTerminator^TM^ Purification Kit (Applied Biosystems^TM^). Electrophoresis was conducted on an ABI 3500xL Genetic Analyzer using POP-7^TM^ polymer, 50 cm capillary array, and default instrument settings (Applied Biosystems^TM^).

We aligned forward and reverse sequences, checked them for quality, and trimmed primer sequences using Sequencher® Version 5.4.6 (Gene Codes Corporation, USA). Resulting trimmed sequences were aligned and compared with references publicly available in GenBank (National Center for Biotechnology Information (NCBI); Ballare et al. 2023) and with in-house sequences from vouchered reference specimens. Sequence variations were identified in Sequencher® version 5.4.6 (Gene Codes Corporation, USA).

For both non-degraded (ND2) and degraded (ND2deg) primers, we produced a single consensus sequence for each of the in-house vouchered reference specimens. The consensus sequence for each unique haplotype and primer pair produced by this study were deposited in GenBank (Table S2). For the two species that had more than a single representative individual, only the red-winged blackbird had two observed unique haplotypes whereas the tricolored blackbird had a single conserved haplotype (Tables S2 and S3). These two observed haplotypes for red-wing blackbirds were represented by a single variation at a single site. In contrast, interspecific differences over the 201 bp region were 10-11 bp for red-winged vs tricolored, and greater for all other species pairs (Table S3).

We also present a nucleotide substitution table for the tricolored blackbird and red-winged blackbird 201 bp ND2 haplotypes observed in this study. Our table also includes the available full-coverage GenBank reference sequences for these two blackbird species (CM041028.1; Ballare et al. 2023, JX516062.1; Powell et al. 2013, KM078767.1; Lerner et al. 2011; MN356439.1; Feng et al. 2020; Table S4).

# SI References

Ballare, K. M., Escalona, M., Barr, K., Seligmann, W., Sacco, S., Sahasrabudhe, R. M., Nguyen, O., Wyckoff, C., Smith, T. B., Shapiro, B. (2023). A reference genome assembly of the declining tricolored blackbird, *Agelaius tricolor*. *The Journal of Heredity*. **114**, 44–51.

Barker, F.K., Benesh, M.K., Vandergon, A.J., Lanyon, S.M. (2012). Contrasting evolutionary dynamics and information content of the avian mitochondrial control region and ND2 gene. *PloS One*. **7**, e46403.

Barker, F.K., Burns, K.J., Klicka, J., Lanyon, S.M., Lovette, I.J. (2013). Going to extremes: contrasting rates of diversification in a recent radiation of new world passerine birds. *Systematic Biology*. **62**, 298–320. <https://doi.org/10.1093/sysbio/sys094>.

Beedy, E.C., Hamilton III, W.J., Meese, R.J., Airola, D.A., Schackwitz, W.S., Pyle, P. (2023). Tricolored Blackbird (*Agelaius tricolor*), version 2.0. In Birds of the World (P. G. Rodewald and B. K. Keeney, Editors). Cornell Lab of Ornithology, Ithaca, NY, USA. <https://doi.org/10.2173/bow.tribla.02>.

Desjardins, P., Morais, R. (1990). Sequence and gene organization of the chicken mitochondrial genome. A novel gene order in higher vertebrates. *Journal of Molecular Biology*. **212**, 599–634.

eBird. (2021). eBird: An online database of bird distribution and abundance [web application]. eBird, Cornell Lab of Ornithology, Ithaca, New York. Available: <http://www.ebird.org>. Accessed 04 June 2022.

Feng, S., Stiller, J., Deng, Y. et al. (2020). Dense sampling of bird diversity increases power of comparative genomics. *Nature*. **587,**252–257.

Johnson, K.P., Lanyon, S.M. (1999). Molecular systematics of the grackles and allies, and the effect of additional sequence (cyt B and ND2). *Auk*. **116**, 759-768.

Lerner, H.R., Meyer, M., James, H.F., Hofreiter, M., Fleischer, R.C. (2011). Multilocus resolution of phylogeny and timescale in the extant adaptive radiation of Hawaiian honeycreepers. *Current Biology.* **21**, 1838–1844.

McCracken, K., Sorenson, M. (2005). Is homoplasy or lineage sorting the source of incongruent mtDNA and nuclear gene trees in the stiff-tailed ducks (*Nomonyx oxyura*). *Systematic Biology.* **54**, 35–55.

Meiklejohn, K.A., Burnham-Curtis, M.K., Straughan, D.J., Giles, J., Moore, M.K. (2021). Current methods, future directions and considerations of DNA-based taxonomic identification in wildlife forensics. *Forensic Science International: Animals and Environments*. **1**, 100030.

Messing, J. (1983). New M13 vectors for cloning. *Methods in Enzymology.* **101**, 20–78.

Powell, A.F., Barker, F.K., Lanyon, S.M. (2013). Empirical evaluation of partitioning schemes for phylogenetic analyses of mitogenomic data: an avian case study. *Molecular Phylogenetics and Evolution*. **66**, 69–79.

**Table S1.** Primers used in this study to amplify and sequence the ND2 gene region for bird species identification. bp = base pairs.

| **Primer** | **Sequence (5’-3’)** | **Amplicon size (bp)** | **Reference** |
| --- | --- | --- | --- |
| M13F | TGTAAAACGACGGCCAGT | - | Messing 1983 |
| M13R | CAGGAAACAGCTATGAC | - | Messing 1983 |
| ND2-L5216 | GGCCCATACCCCGRAAATG | - | McCracken and Sorenson 2005 |
| ND2-H5766 | RGAKGAGAARGCYAGGATYTTKCG | 549-550 | McCracken and Sorenson 2005 |
| ND2-H5418 | GCAGCTGCYTGGGTTAAGAA | 201 | This study |

**Table S2.** Avian reference specimens used in this study and their associated GenBank accession numbers. Footnotes refer to the sources for the in-house reference samples used in this study. ND2 refers to the full consensus sequence for non-degraded DNA; ND2deg refers to the full consensus sequence used when DNA was degraded.

| **Common name (species)** | **Number of representative individuals** | **Number of haplotypes observed** | **GenBank accession numbers** | |
| --- | --- | --- | --- | --- |
|  |  |  | **ND2** | **ND2deg** |
| Red-winged blackbird (*Agelaius phoeniceus*) ^1^ | 5 | 2 | PQ490372-PQ490376 | PQ490384-PQ490388 |
| Tricolored blackbird (*Agelaius tricolor*) ^2^ | 3 | 1 | PQ490377-PQ490379 | PQ490389-PQ490391 |
| Rusty blackbird (*Euphagus carolinus*) ^3^ | 1 | 1 | PQ490380 | PQ490392 |
| Brewer's blackbird (*Euphagus cyanocephalus*) ^1^ | 1 | 1 | PQ490381 | PQ490393 |
| Brown-headed cowbird (*Molothrus ater*) ^4^ | 1 | 1 | PQ490382 | PQ490394 |
| Yellow-headed blackbird (*Xanthocephalus xanthocephalus*) ^5^ | 1 | 1 | PQ490383 | PQ490395 |

*^1^ California Department of Fish and Wildlife (CDFW) Law Enforcement Division.*

*^2^ CDFW California Endangered Species Act (CESA) Conservation Unit.*

*^3^ University of Alaska Museum Bird Collection specimen UAM 36539.
^4^ CDFW Wildlife Health Laboratory (WIL).
^5^ University of California, Berkeley, Museum of Vertebrate Zoology specimen MVZ 182330.*

**Table S3.** Correlation matrix for sequence haplotypes among and within species. Numerical values indicate the observed base pair (bp) differences over the 201 bp ND2 gene region when the reference specimen haplotypes are compared.

| **Species** | **Red-winged blackbird** | **Tricolored blackbird** | **Rusty blackbird** | **Brewer's blackbird** | **Brown-headed cowbird** | **Yellow-headed blackbird** |
| --- | --- | --- | --- | --- | --- | --- |
| **Red-winged blackbird** | 0-1 | - | - | - | - | - |
| **Tricolored blackbird** | 10-11 | 0 | - | - | - | - |
| **Rusty blackbird** | 23-24 | 21 | 0 | - | - | - |
| **Brewer's blackbird** | 20-21 | 17 | 11 | 0 | - | - |
| **Brown-headed cowbird** | 18-19 | 16 | 20 | 18 | 0 | - |
| **Yellow-headed blackbird** | 21-22 | 20 | 22 | 20 | 23 | 0 |

**Table S4.** Nucleotide substitution table for observed base pair differences over the 201 bp ND2 gene region for the observed red-winged blackbird and tricolored blackbird reference specimen haplotypes. Corresponding nucleotide basepair positions are in accordance with the tricolored blackbird mitochondrial genome CM041028.1. Red-winged blackbird mitochondrial sequences were also included for comparison. Only GenBank reference sequences that provided entire coverage for the sequence comparison alignment region were included. An asterisk (“*”) represents the same nucleotide for the indicated position as for CM041028.1. A colon (“:”) represents a gap in the sequence coverage for the indicated nucleotide position. “N” represents an ambiguous base for at the indicated nucleotide position.

|  | 4,032 | 4,037 | 4,054 | 4,064 | 4,070 | 4,079 | 4,079.1 | 4,080 | 4,081.1 | 4,082 | 4,084.1 | 4,088 | 4,115 | 4,154 | 4,178 |
| --- | --- | --- | --- | --- | --- | --- | --- | --- | --- | --- | --- | --- | --- | --- | --- |
| **Tricolored blackbird** |  |  |  |  |  |  |  |  |  |  |  |  |  |  |  |
| CM041028.1 | A | C | T | T | T | A | : | A | : | T | : | C | C | G | T |
| TRBL haplotype 1 | * | * | C | * | * | * | * | * | * | * | * | * | * | * | * |
| **Red-winged blackbird** |  |  |  |  |  |  |  |  |  |  |  |  |  |  |  |
| NC_018801.1 | G | T | C | C | * | G | * | * | * | C | * | T | T | A | C |
| JX516062.1 | G | T | C | C | * | G | * | * | * | C | * | T | T | A | C |
| KM078767.1 | G | T | C | C | C | G | G | * | N | C | T | T | T | A | C |
| MN356439.1 | G | T | C | C | C | G | * | * | * | C | * | T | T | A | C |
| AGPH haplotype 1 | G | T | C | C | * | G | G | : | * | C | * | T | T | A | C |
| AGPH haplotype 2 | G | T | C | C | C | G | G | : | * | C | * | T | T | A | C |

**Fig. S1.** Annual and monthly (temporal) patterns in uncorrected (raw) numbers of fatalities of blackbirds and cowbirds at the geographic regions of the Altamont Pass Wind Resource Area, California, USA from 21 publicly available reports and datasets documenting individual fatality observations from 2005 – 2023 (the report covering 1998 -2003 is used in other analyses but did not specify monthly totals and so was not included here). Plots show (A) species-specific annual count of reported blackbird fatalities; (B) species-specific monthly count of fatalities across all sampling years; and (C) and (D) updated annual count of fatalities originally given in (A) and the monthly count of fatalities originally given in (B), respectively, with the additional information provided by genetic identification for species originally classified as “unidentified blackbird” (*n* = 26) or misidentified as another blackbird species (*n* = 2). Species include brown-headed cowbird (BHCO), and Brewer’s (BRBL), red-winged (RWBL), tricolored (TRBL), and unidentified (UNBK) blackbirds.

**
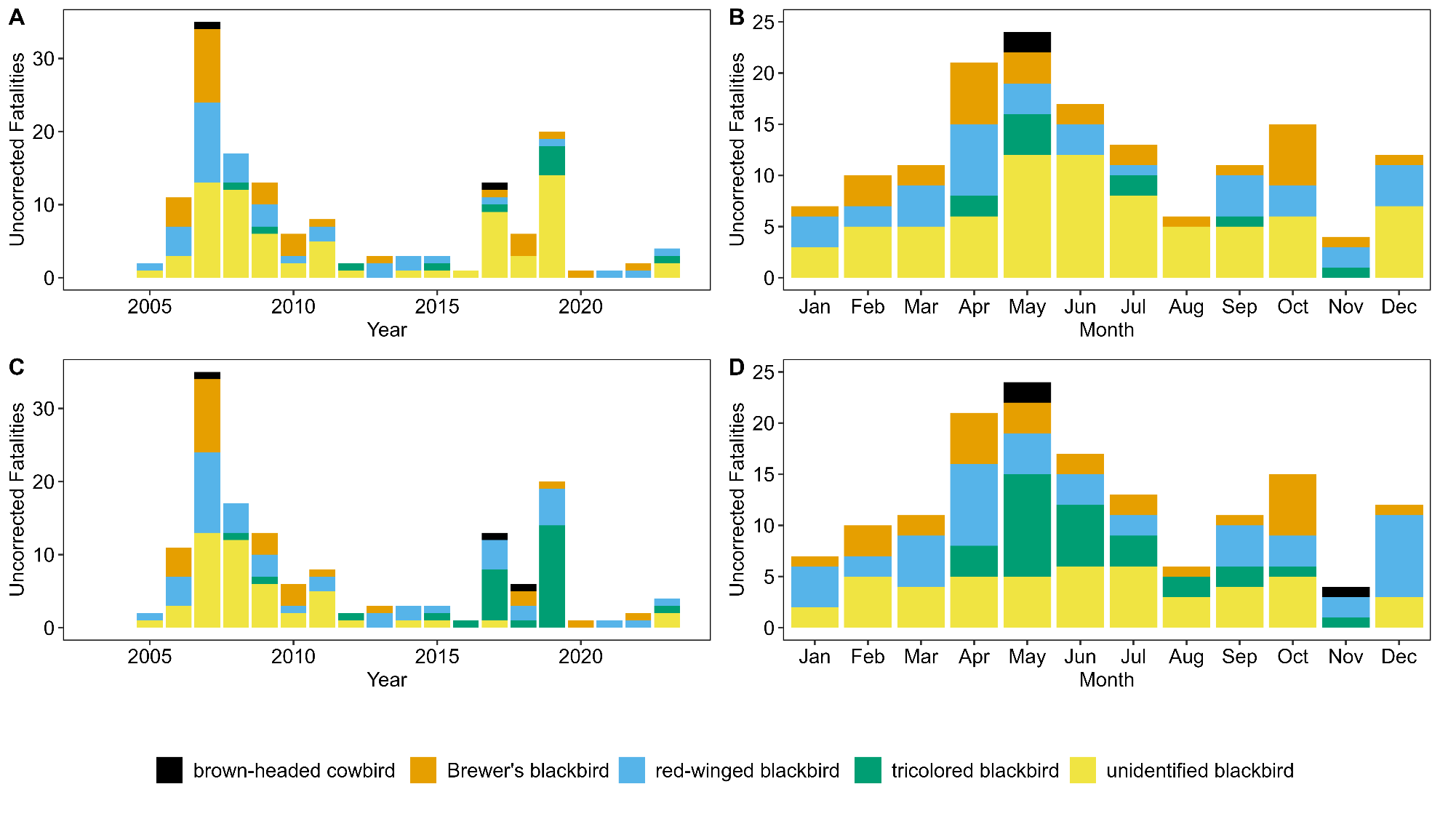
**
